# Influenza B virus has global ordered RNA structure in (+) and (−) strands but relatively less stable predicted RNA folding free energy than allowed by the encoded protein sequence

**DOI:** 10.1186/1756-0500-6-330

**Published:** 2013-08-19

**Authors:** Salvatore F Priore, Walter N Moss, Douglas H Turner

**Affiliations:** 1 Department of Chemistry and Center for RNA Biology, University of Rochester, Rochester, NY 14627-0216, USA

**Keywords:** RNA, RNA secondary structure, Influenza, Influenza A, Influenza B, Structural bioinformatics, GORS

## Abstract

**Background:**

Influenza A virus contributes to seasonal epidemics and pandemics and contains Global Ordered RNA structure (GORS) in the nucleoprotein (NP), non-structural (NS), PB2, and M segments. A related virus, influenza B, is also a major annual public health threat, but unlike influenza A is very selective to human hosts. This study extends the search for GORS to influenza B.

**Findings:**

A survey of all available influenza B sequences reveals GORS in the (+) and (−)RNAs of the NP, NS, PB2, and PB1 gene segments. The results are similar to influenza A, except GORS is observed for the M1 segment of influenza A but not for PB1. In general, the folding free energies of human-specific influenza B RNA segments are less stable than allowable by the encoded amino acid sequence. This is consistent with findings in influenza A, where human-specific influenza RNA folds are less stable than avian and swine strains.

**Conclusions:**

These results reveal fundamental molecular similarities and differences between Influenza A and B and suggest a rational basis for choosing segments to target with therapeutics and for viral attenuation for live vaccines by altering RNA folding stability.

## Findings

### Introduction

In contrast to influenza A, a zoonotic pathogen that infects multiple host species, influenza B primarily infects humans and, rarely, seals [1,2]. Influenza B also differs from influenza A by having a lower mutation rate and fewer antigenic serotypes [3]. Though its lack of antigenic diversity bars pandemic outbreaks, influenza B contributes to seasonal occurrences of influenza, which can result in serious infections costing thousands of lives and billions of dollars [4,5]. Influenza B has been of increasing concern lately, due to the rise in circulation of two distinct lineages of the virus: Victoria and Yamagata, which stimulated the recent switch from a trivalent vaccine (against one influenza B and two influenza A serotypes) to a quadrivalent vaccine including both influenza B serotypes [6,7]. The viral genome is comprised of eight negative sense, or (−)RNA, segments. Segments NS, M1/BM2, and NA encode multiple protein products via alternative initiation, termination-reinitiation, and splicing, respectively [8].

RNA secondary structure plays important roles in the biology of many viruses: for example, in gene expression [9], splicing [10], molecular stability/life-time [11], and control of host gene expression [12]. Some RNAs, such as compact viral genomes, can encode both protein information and functional RNA secondary structures [13]. The importance of RNA structure in influenza virus protein coding regions, or (+)RNA, is now being revealed. For influenza A, structures have been described towards the 5′ end [14] and at the 3′ splice site [15,16] of segment NS (+)RNA. Both structures may have a role in the regulation of splicing. When many sequences are available, predicted folding stabilities can identify RNA regions likely to have structure [17]. A survey of all influenza A coding sequences found evidence for multiple sites with probable locally conserved RNA structure in the (+)RNA [18]. Similar to segment NS, structures were discovered in the 5′ region and 3′ splice site of segment M. The structure at the 3′ splice site can switch between pseudoknot and hairpin conformations, respectively, burying or revealing the splice site and other splicing signals [19]. Thus, this structure may have a role in regulation of segment M splicing.

In addition to locally conserved RNA structure, a survey of all influenza A sequences revealed global ordered RNA structure (GORS) that extends throughout (+) and (−) RNA for the NP, NS, PB2, and M1 genes (an error in our previous calculations of GORS in influenza A (−)RNA [20] gave the incorrect result that this orientation lacked conserved structure. Correction of this mistake revealed that genes with GORS in the (+)RNA also possessed GORS in the (−)RNA. GORS is revealed by predicting “excess” thermodynamic stability of wild-type RNA sequences versus random RNA of the same composition, as represented by a z-score [21]:

(1) $$z‒\text{score}=\frac{\left(\Delta G_{\left(37,\mathrm{wld}-\text{type}\right)}^{o}-\mu \right)}{\sigma }$$

Here ΔG°_37, wild-type_ is the predicted folding free energy of the wild-type sequence, μ is the average predicted folding free energy of the dinucleotide randomizations, and σ is the standard deviation of the randomized population. GORS is defined as a significant negative shift in the median z-score away from an ideal non-structured RNA population (i.e. a normal distribution centered at zero). Thus, segments with a median z-score below −0.67 are considered to have GORS.

While free energy minimization has limited accuracy and, in most algorithms, forbids pseudoknots [22], it can on average correctly predict roughly 73% of base pairs [23]. Estimating free energies is an easier problem. For example, structures with greater than 86% of correctly predicted base pairs typically differ from the minimum free energy structure by an average of only 5% in their ΔG°_37_ values [24]. Thus, good estimations of the relative thermodynamic stability within the same segment and between wild-type and matched randomized controls is achievable.

Many RNA viruses have negative shifts in z-scores for (+)RNAs relative to unstructured sequences [25,26], implying widespread RNA structure. Studies in bacterial mRNAs found similar patterns [27]. Influenza A has GORS in both orientations of the NP, NS, PB2, and M gene segments. Generally in influenza A, avian strains are the most stable, followed by swine and then human [20]. A similar trend was found for the z-scores of NP, NS, and PB2 gene segments. The exact role of GORS is unclear, but may be a mechanism for evasion of the host innate immune system [25] or for controlling mRNA life-time/stability [28]. Identification of segments with and without GORS could help guide discovery of targets for small molecules and oligonucleotide therapeutics against influenza virus, since these approaches require structured and unstructured RNA targets, respectively.

This study extends to influenza B the search for global trends in RNA structure. Because only human influenza B strains are available, the folding free energies and z-scores of influenza B sequences are compared to folding free energies and z-scores of synonymous codon mutations (i.e. sequences that code for the same protein as wild-type influenza B sequences) generated *in silico*. Additional comparisons are made between results for influenza A and B. Similarities and differences are observed, which imply that influenza B has a distinctly different biology from influenza A.

### Materials and methods

The research in our lab, including the content of this manuscript, has been performed with the approval of the University of Rochester’s research ethics committee.

Coding regions for all unique influenza B mRNAs were downloaded from the NCBI Influenza Virus Resource Page [29]. Truncated sequences or those with ambiguous nucleotides were removed, leaving 4110 sequences: 370 in NP, 519 in NS, 363 in PB2, 339 in PB1, 350 in M1, 832 in HA, 354 in PA, and 983 in NA. RNA folding free energies for the entire coding regions were predicted by minimizing the ΔG°_37_ with the program RNA fold [30]. Z-scores [21] were calculated for all sequences by comparing the free energy of wild-type sequences to sets of ten randomized sequences, which preserved dinucleotide content using the Simmonics Sequence Editor [31,32]. A negative z-score implies GORS [20]. In this work, a population of single sequences with a median z-score below −0.67 is considered to possess GORS. We will apply the same definition to a reanalysis of our previous results for influenza A [20].

To generate sets of synonymous codon mutants for comparison with folding free energies and z-scores of wild-type sequences, one coding region for each of the eight segments was mutated *in silico* to produce eight sets of 500 synonymous mutant sequences. Five hundred randomizations of one sequence from each segment was considered sufficient because the protein sequences are ~100% conserved in the available influenza B sequences. Synonymous codon mutations were made with a PERL script that randomly selected codons and made synonymous substitution at those sites, including substituting the same codon (no change). Folding free energy and z-scores were calculated as described above for wild-type. Specifically, ten dinucleotide randomizations of each of the 500 synonymous codon mutants were used for calculating 500 z-scores for each influenza B segment.

Box plots were constructed for each population of predicted free energies and z-scores. The box on each plot represents the interquartile range (IQR) which is defined as the difference between the 75th percentile (Q3) and 25th percentile (Q1) of each population. Upper and lower bounds for each plot (bars extending from the box) represent the largest and smallest data values within 1.5 × IQR of the Q3 and Q1, respectively. Values outside of this area are considered anomalous for that population.

### Results

**Figure 1:**
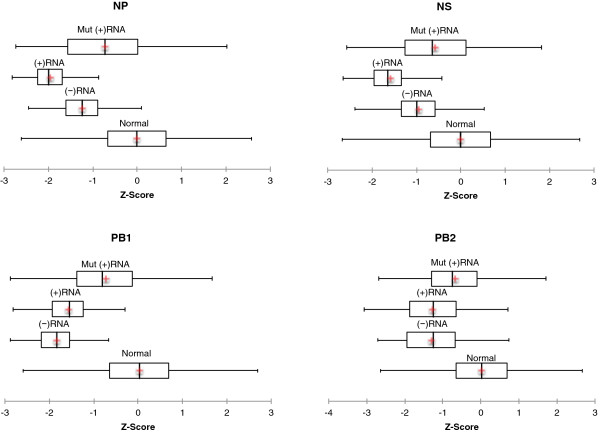
**Box plots of z-scores for influenza B wild-type coding regions with evidence of global ordered RNA structure and mutant sequences coding for the same protein: Boxes represent the interquartile region (IQR = Q**_**3**_**– Q**_**1**_**) for each distribution.** The left edge of the box is the 25th percentile (Q_1_) and the right edge is the 75th percentile (Q_3_). The bar inside the box indicates the median and the red cross indicates the mean. Bars extending from the right and left of the box indicate the upper and lower bounds, respectively (See Materials and methods). GORS is considered present when the median for influenza sequences lies outside the IQR expected for an unstructured control population (i.e. a normal distribution centered at zero) shown at the bottom of each plot.

**Figure 2:**
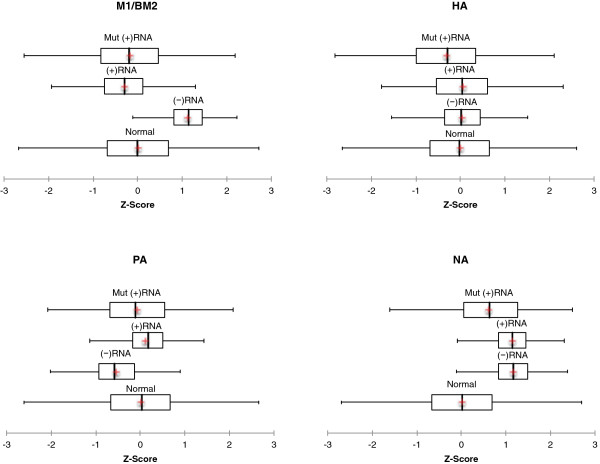
**Box plots of z-scores for influenza B wild**-**type coding regions and mutant sequences coding for the same protein with no evidence of global ordered RNA structure: see Figure **1** for annotations and details.**

Clear evidence for influenza B GORS is found in the (+) and (−) strands of segments NP, NS, PB2, and PB1, with NP having the most favorable median z-score (Table 1). Distributions of z-scores for these sequences were almost entirely in the negative region (Figure 1 and Additional file 1: Figure S1). The remaining coding regions have average z-scores close to zero or positive (Table 1). The z-score distributions for the sequences that did not show GORS generally centered near zero or trended towards the positive (Figure 2 and Additional file 2: Figure S2).

**Table 1:** Median z-scores and average predicted folding free energy for influenza B (+)RNA, (−)RNA and synonymous codon mutant (mut (+)RNAs)

**Segment**		**Z-score (+)RNA**	**Z-score Mut (+)RNA**	**Z-score (−)RNA**	**ΔG°**_**37**_**(+)RNA**	**ΔG°**_**37**_**Mut (+)RNA**	**ΔG°**_**37**_**(−)RNA**
5	NP	−2.0	−0.7	−1.2	−494.5	−493.5	−485.7
8	NS	−1.6	−0.6	−1.0	−279.4	−283.1	−264.5
2	PB1	−1.6	−0.8	−1.8	−601.7	−624.1	−580.9
1	PB2	−1.3	−0.7	−1.2	−600.6	−638.2	−538.2
7	M1/BM2	−0.3	−0.2	1.1	−276.6	−290.8	−245.6
4	HA	0.0	−0.3	0.0	−485.1	−511.4	−506.7
3	PA	0.2	−0.1	−0.6	−552.0	−593.0	−501.3
6	NA	1.1	0.6	1.2	−384.2	−409.5	−374.8

Negative z-scores below −0.67 reveal GORS.

**Figure 3:**
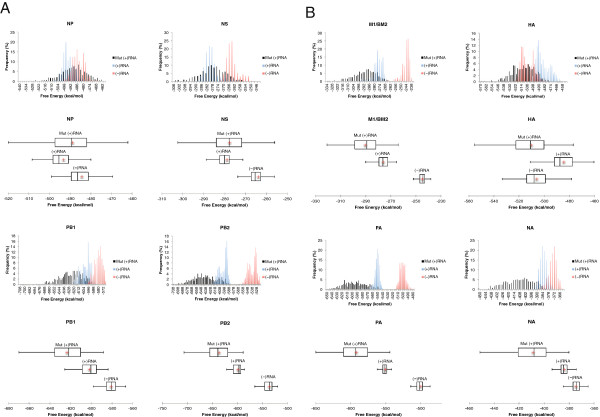
**Box plots and predicted minimum free energy distributions** (**ΔG**°_**37**_**in kcal**/**mol**) **for influenza B coding regions with (A) and without (B) GORS.** Predicted free energy distributions are shown in the first, third, fifth, and seventh rows. (+)RNA, (−)RNA, and synonymous codon mutant (+)RNA are colored blue, red and black, respectively. Box plots are shown below their corresponding predicted free energy distribution. Predicted free energy in kcal/mol at 37°C is reported on the x-axis. For predicted free energy distribution plots, bins are in 1 kcal/mol increments. Percentages of sequences in each bin are reported on the y-axis. Bars extending from the right and left of the box indicate the upper and lower bounds, respectively (See Materials and methods). Distributions are considered well separated from each other if their IQRs do not overlap.

With the exception of HA, distributions of predicted free energies for influenza B are shifted towards more stability in the (+)RNA versus the (−)RNA (Figure 3), so (+)RNAs have more favorable predicted average folding free energies than (−)RNAs (Table 1). Free energy of folding also favored the (+)RNA for all segments in influenza A [20].

Unlike influenza A, there are no avian or swine sequences available to compare the relative predicted stabilities of folding in other species for each segment of influenza B. To simulate this comparison, sets of synonymous codon mutants were generated. The *in silico* synonymous codon mutant sets provide distributions of free energies for each influenza B coding region where the only constraint is to maintain the encoded protein product. They thus represent the potential RNA folding free energy landscape allowed by the encoded amino acid sequence. Predicted ΔG°_37_ indicates that wild-type sequences in the (+)RNA sense generally have less stable secondary structure than sequences with codon mutants (Table 1). Only NP breaks this trend, where the *in silico* (+)RNA mutants are on average less stable by 1.0 kcal/mol at 37°C. Distributions of free energies for the mutant sequences have greater spread than wild-type sequences and are also generally shifted towards more favorable thermodynamic stability versus the wild-type sequences (Figure 3). Evidently, the average thermodynamic stability of wild-type sequences is less favorable than allowed by protein coding constraints, even though global RNA structure is present in at least four coding regions. The wild-type sequences occupy a small part of the range of free energies allowed by the amino acid sequence and are distributed towards less favorable stability (Figure 3). An examination of nucleotide frequencies reveals that synonymous codon mutants have at least 2% higher GC content than wild-type sequences (Table 2).

**Table 2:** Average GC content of wild-type (+)RNA influenza B sequences and synonymous codon mutant sequences

**Segment**	**Avg. % GC wild-type (+)RNA**	**Avg. % GC Mut (+) RNA**
5 NP	43	45
8 NS	41	43
2 PB1	39	42
1 PB2	39	42
7 M1/BM2	40	42
4 HA	43	45
3 PA	39	44
6 NA	45	47

Z-scores were also calculated for the synonymous codon mutant sets. Compared to distributions of the four wild-type sequences with evidence of GORS, all but the NS segment mutants still possess GORS. In the three cases, however, the median z-scores for mutants were more positive than for wild-type sequences (Table 1, Figures 1 and 2).

### Discussion

Predictions of GORS can partition RNA sequences into regions with or without strong secondary structure. Such partitioning should be helpful in identifying regions easier to target with therapeutics. For example, small molecules will bind specifically to structured regions, whereas oligonucleotide based therapeutics will bind more tightly to unstructured regions. Prediction of regions with GORS may also facilitate genome-wide probing of secondary structure [33-35] by focusing searches to regions likely to have conserved structure.

For influenza B, three of the four gene segments with GORS have homologs in influenza A that also show GORS [20]: NP, NS, and PB2. Unlike influenza A, there is no evidence for GORS in the influenza B M1/BM2 gene. A possible explanation for this lack of GORS is that in influenza A, segment M encodes both the M1 (matrix protein) and M2 (ion channel) proteins, which are alternatively spliced, whereas in influenza B the BM2 open reading frame directly follows M1 and is translated via termination-reinitiation [36,37]. In influenza A, local RNA structures have been described that have implications for splicing [15,18,19]. Perhaps GORS is absent in influenza B M1/BM2 because there is no need for RNA structures important for splicing.

In influenza B, the PB1 coding region shows strong evidence of GORS (median z-score of −1.5), in contrast to influenza A where the average z-scores are equal to or more positive than −0.5 [20]. This suggests PB1 of influenza B must maintain structure to stabilize mRNA for some yet unknown reason that is not present for influenza A PB1. Interestingly, the (−)RNA z-score for this region is more favorable than the (+)RNA. This suggests an important role for structure in the genomic RNA for this segment, with structure in the (+)RNA representing a structural “echo”.

The result of less favorable relative thermodynamic stability of influenza B sequences when compared with a set of randomly generated synonymous codon sequences is consistent with the human host species specificity of influenza B. For influenza A, sequences specific to humans have less favorable thermodynamic stability than swine and avian species, even though protein sequence is largely conserved [20]. However, any changes in thermodynamic stability in synonymous codon mutants for all segments appears to be independent of GORS because the average z-score for the mutants was close to zero. A decrease of CpG dinucleotide frequencies in human influenza viruses has been established [38]. As seen in Table 2, synonymous codon mutants acquired increased GC content, which increased their predicted thermodynamic stability, compared to wild-type sequences. This is consistent with the increased GC content of avian influenza A strains compared to human influenza A strains [39]. It appears that evolution, acting to reduce CpG frequency or other factors related to the human host, restricts the thermodynamic stability of influenza B sequences to a small portion of the available folding landscape. Thus, this thermodynamic difference may distinguish human-adapted influenza strains from strains that replicate in other host species.

This work elucidates some of the thermodynamic and structural constraints that may be acting on influenza B RNA sequences and human influenza viruses in general. Some characteristics are shared between influenza B and A: GORS is seen in NS, NP, and PB2 RNAs of both viral species. With the exception of influenza B HA, ΔG°_37_ favors folding in the (+)RNA over the (−)RNA, and the human-specific wild-type influenza B sequences have less favorable thermodynamic stability than allowed by the amino acid sequence. This latter trend was also seen in human influenza A viruses when compared to swine and avian strains [20]. Differences with influenza A are also apparent: For influenza B, the PB1 RNA shows GORS, while influenza A has GORS in the M gene segment. These results imply differences in the role of RNA folding in the two viral groups. A better understanding of the constraints acting on influenza B sequences may aid in the rational attenuation of viral strains for use in vaccines, as has been recently shown with the influenza B NP segment [40].

### Availability of supporting data

The data supporting the results of this article are included within the article (and its additional files).

## Abbreviations

GORS: Global ordered RNA structure.

## Competing interests

The authors declare that they have no competing interests.

## Authors’ contributions

WNM and SFP jointly conceived the experiments, collected and analyzed the data, and wrote the manuscript. DHT analyzed the results and helped write and revise the manuscript. All authors read and approved the final manuscript.

## Supplementary Material

Additional file 1: Figure S1 Frequency distributions (in percent) of z-scores for influenza coding regions with evidence of global ordered RNA structure: top, middle, and bottom rows are for the (+)RNA, (−)RNA, and synonymous codon mutant (+)RNA, respectively. Click here for file

Additional file 2: Figure S2 Frequency distributions (in percent) of z-scores for influenza coding regions with no evidence of global ordered RNA structure: top, middle, and bottom rows are for the (+)RNA, (−)RNA, and synonymous codon mutant (+)RNA, respectively. Click here for file
