# Supplementary figures and images for: Influenza B virus has global ordered RNA structure in (+) and (−) strands but relatively less stable predicted RNA folding free energy than allowed by the encoded protein sequence
Source: BMC Res Notes. 2013 Aug 19;6:330. doi: 10.1186/1756-0500-6-330 (PMC3765861; doi:10.1186/1756-0500-6-330)

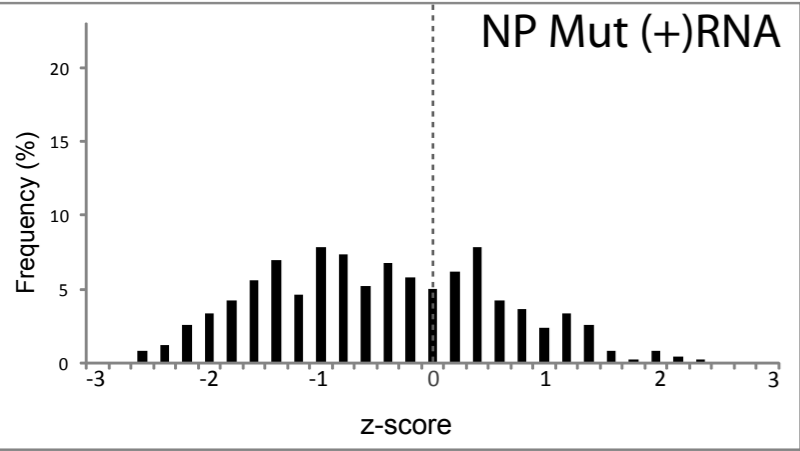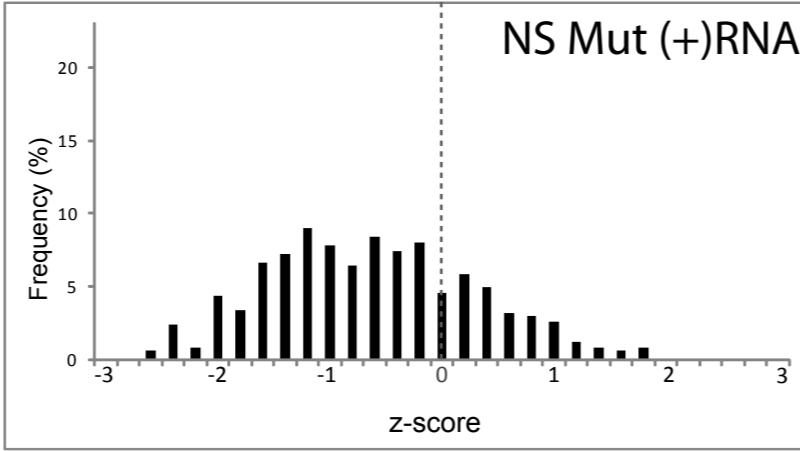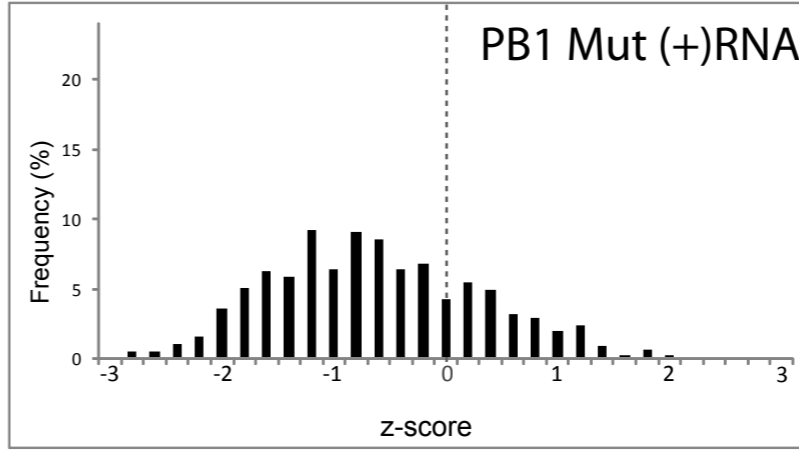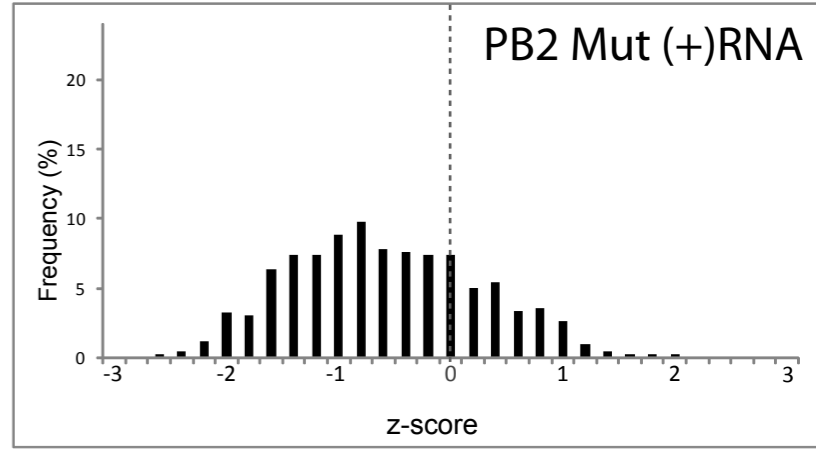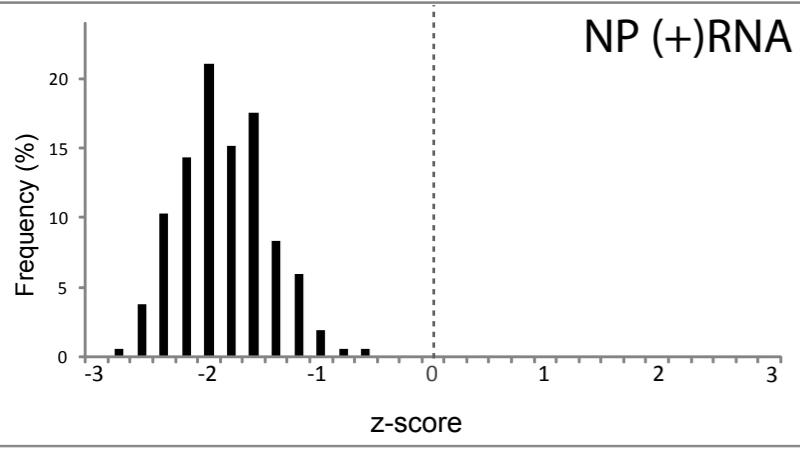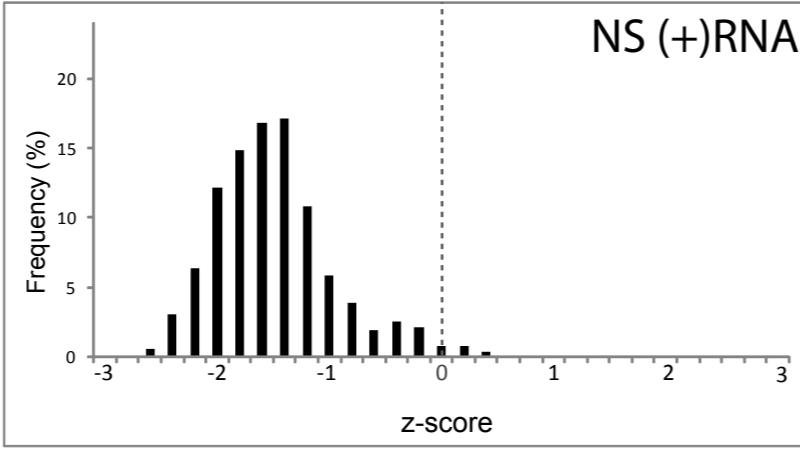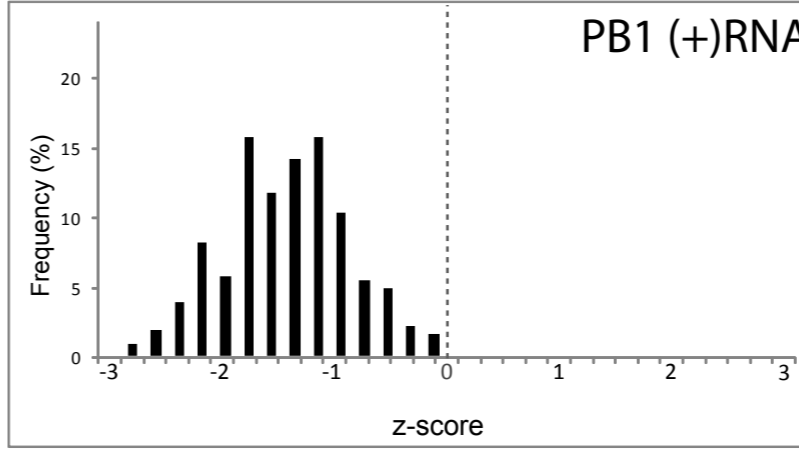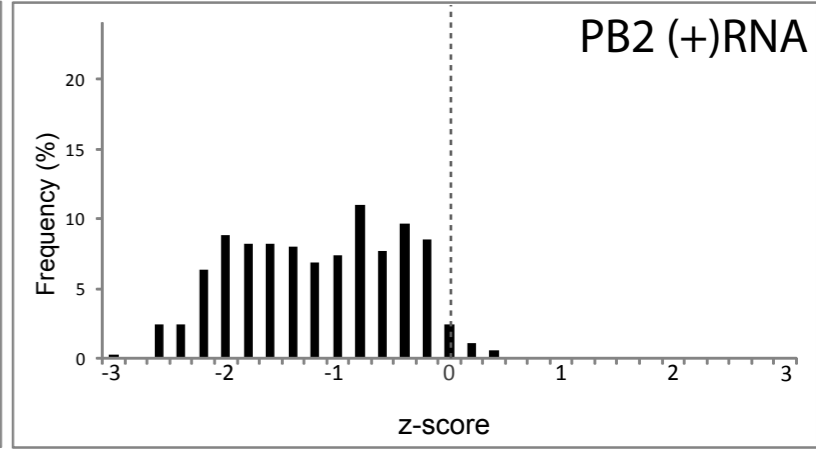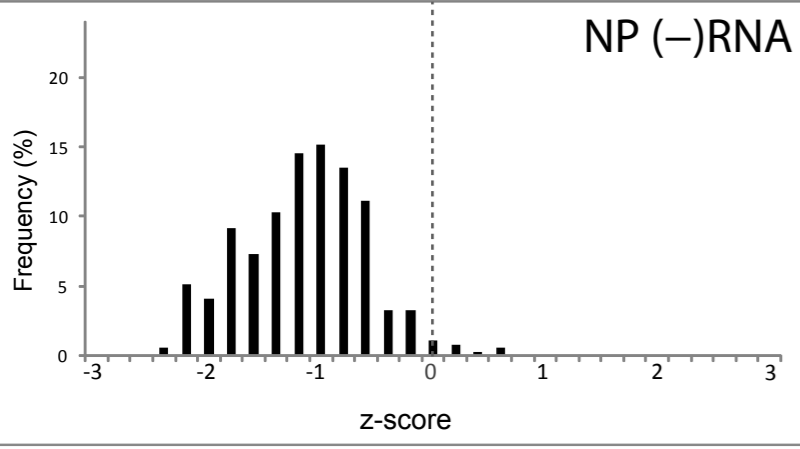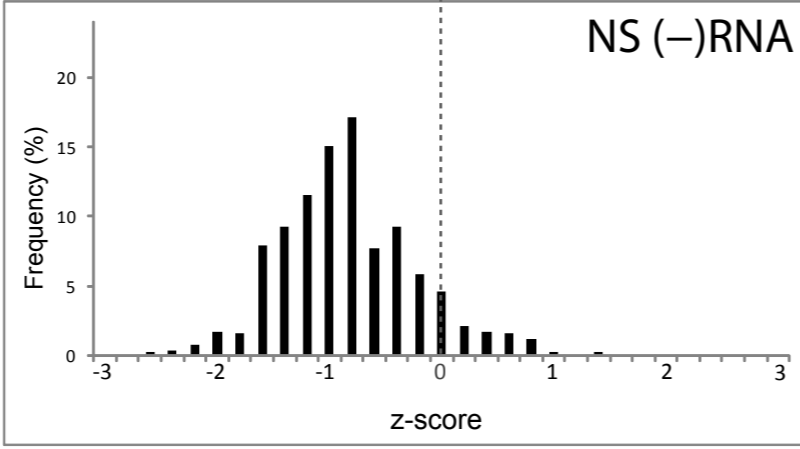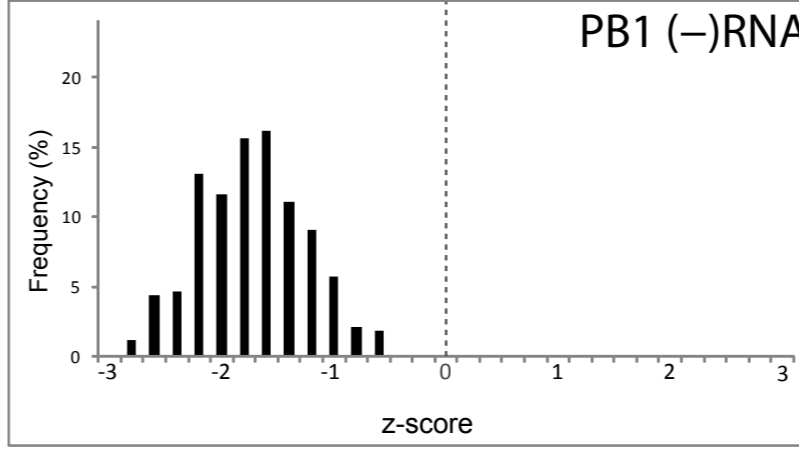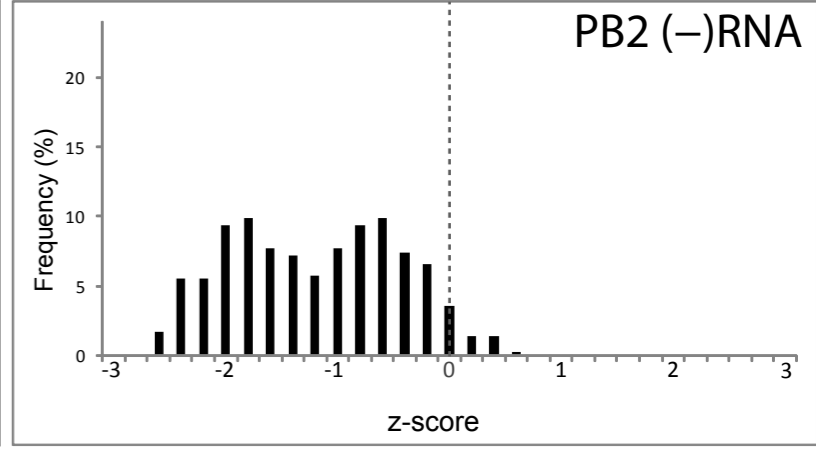

Supplement: Additional file 1: Figure S1 — Frequency distributions (in percent) of z-scores for influenza coding regions with evidence of global ordered RNA structure: top, middle, and bottom rows are for the (+)RNA, (−)RNA, and synonymous codon mutant (+)RNA, respectively. [file 1756-0500-6-330-S1.pdf]

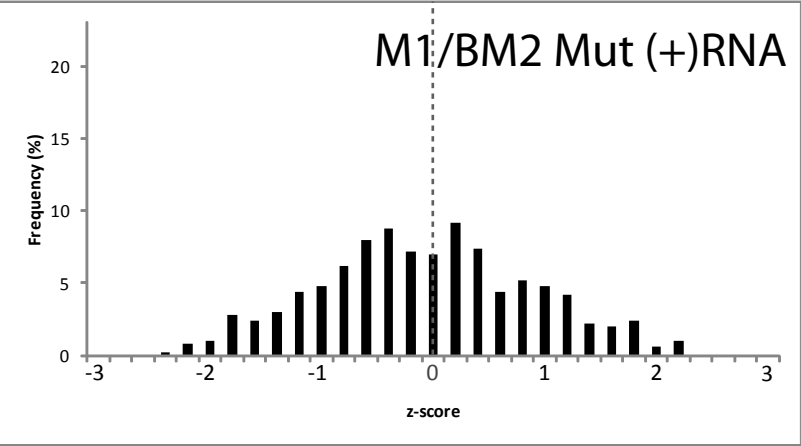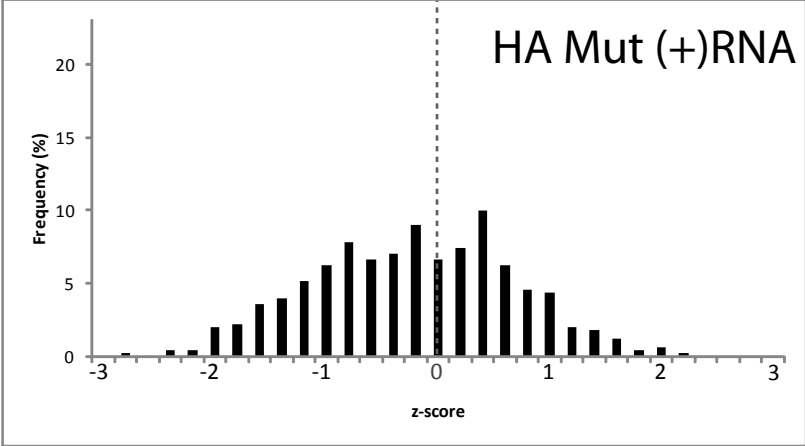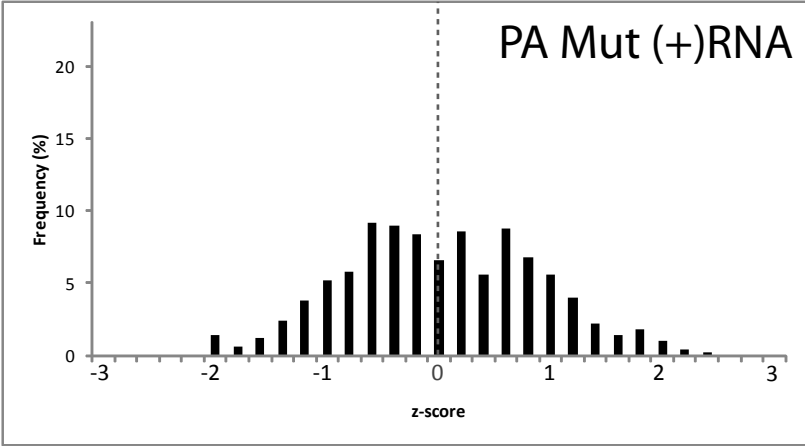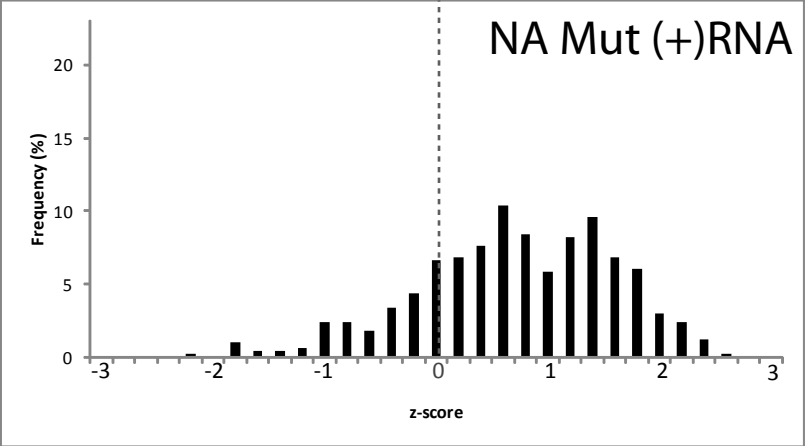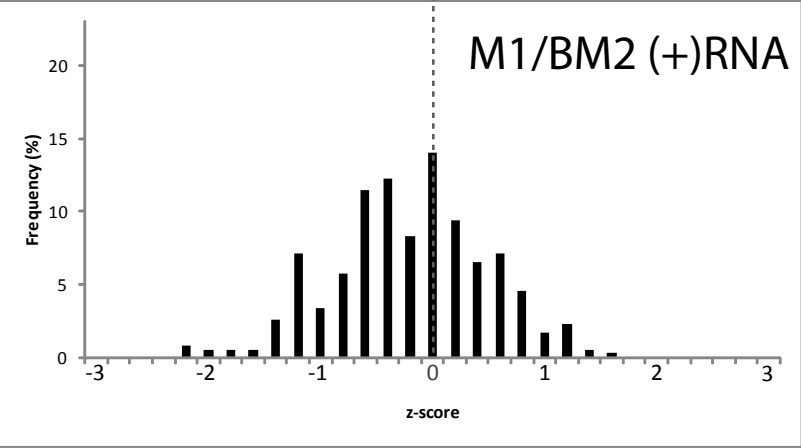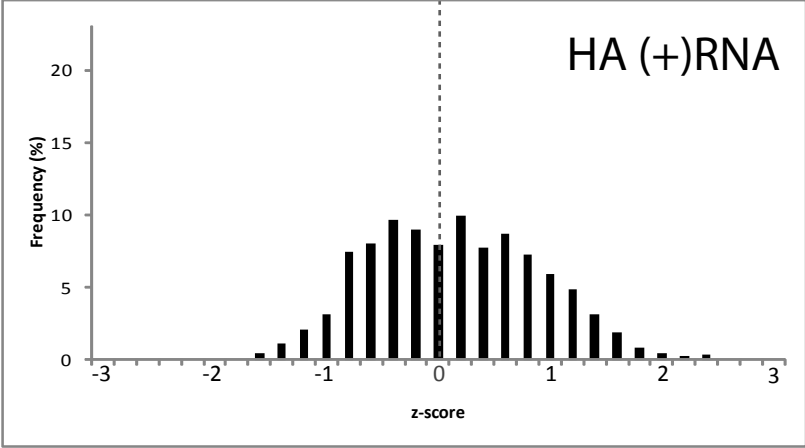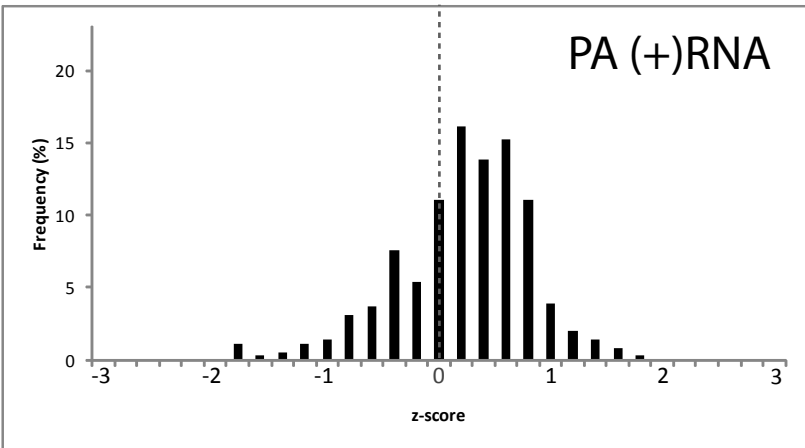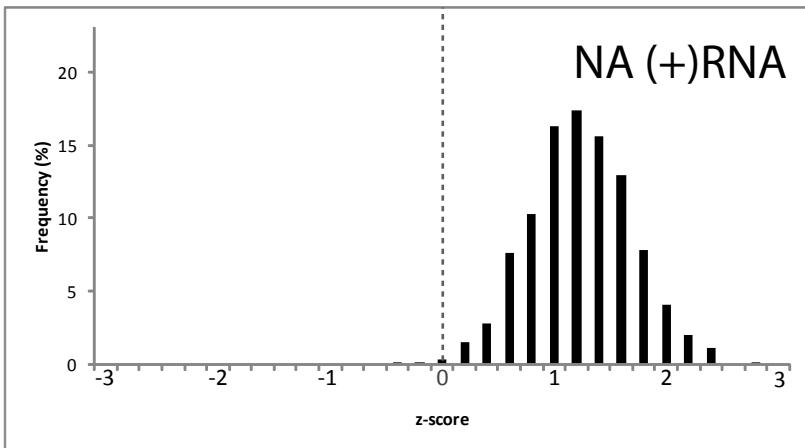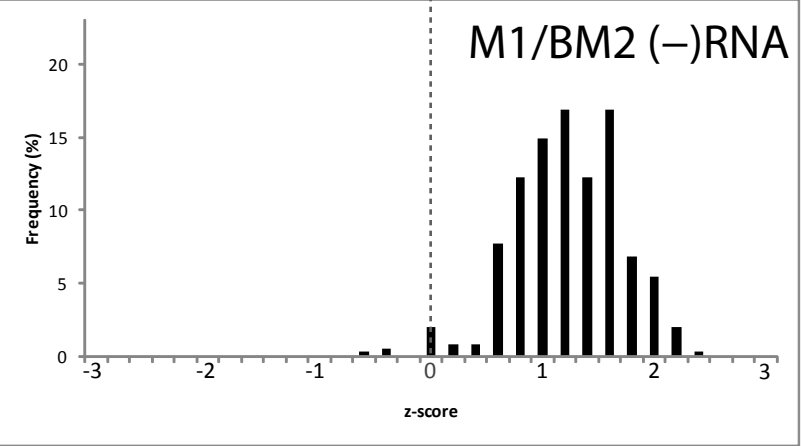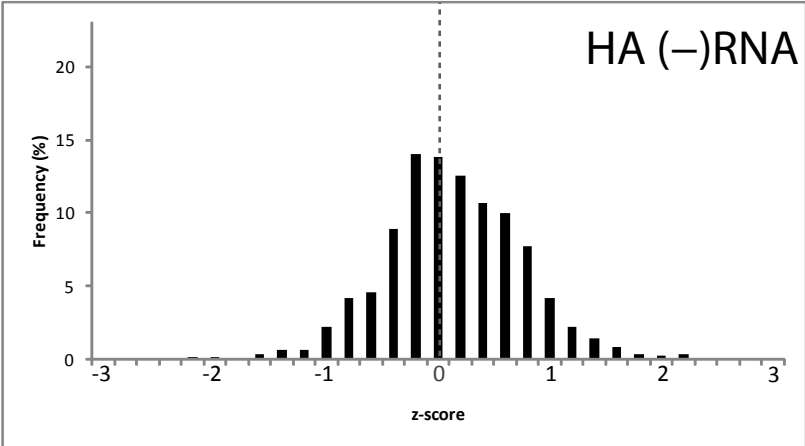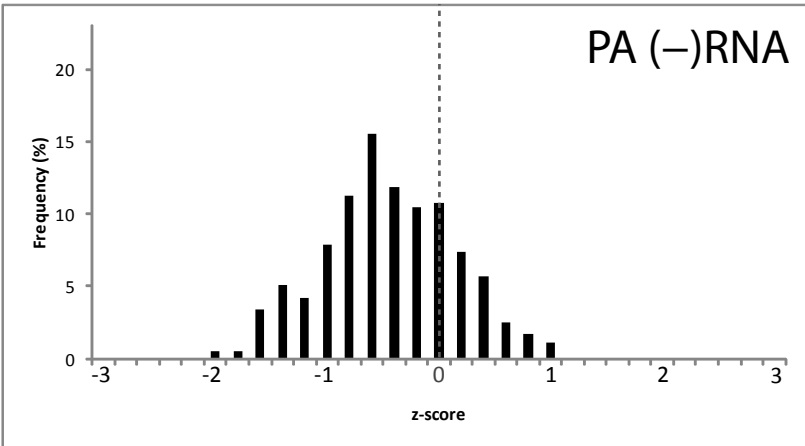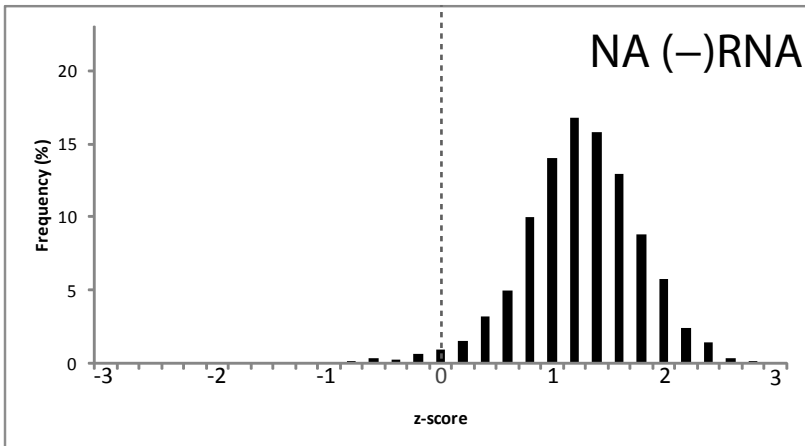

Supplement: Additional file 2: Figure S2 — Frequency distributions (in percent) of z-scores for influenza coding regions with no evidence of global ordered RNA structure: top, middle, and bottom rows are for the (+)RNA, (−)RNA, and synonymous codon mutant (+)RNA, respectively. [file 1756-0500-6-330-S2.pdf]
